# Stimulation of adventitious root formation by laser wounding in rose cuttings: A matter of energy and pattern

**DOI:** 10.3389/fpls.2022.1009085

**Published:** 2022-09-29

**Authors:** Raul Javier Morales-Orellana, Traud Winkelmann, Andreas Bettin, Thomas Rath

**Affiliations:** ^1^ Hochschule Osnabrück - University of Applied Sciences, Biosystem Engineering Laboratory (BLab), Osnabrück, Germany; ^2^ Leibniz Universität Hannover, Institute of Horticultural Production Systems, Section Woody Plant and Propagation Physiology, Hannover, Germany

**Keywords:** adventitious root formation, histology, laser ablation, *Rosa canina*, stem cutting, wounding

## Abstract

Adventitious root (AR) formation is the basis of vegetative propagation in rose, be it *via* stem cuttings or *via* stenting. During this process, wounding plays a pivotal role since cell reprogramming takes place at the tissue adjacent to the wound. We investigated the effects of wounding on AR formation on leafy single-node stem cuttings of the rose rootstock *R. canina* ‘Pfänder’ (codes R02-3 and R02-6) and the cut rose cultivar *Rosa* ‘Tan09283’ (Registration name ‘Beluga’). Laser wounding treatments were based on the assisted removal of tissue layers located in the bark. The positioning of wounding was studied based on two marking directions: along the cutting base (strip pattern) and around the cutting base (ring pattern). Additionally, the effects of external supply of indole-butyric acid (IBA 1 mg L^-1^) on rooting were analyzed. Results showed that in order to remove specific tissue layers, the calculation of the laser energy density (J cm^-2^) in terms of cutting diameter was necessary. Interestingly, the application of energy densities from 2.5 J cm^-2^ up to approximately 8.5 J cm^-2^ were sufficient to expose the tissue layers of epidermis up to regions of phloem. Regarding AR formation for *R. canina* ‘Pfänder’, characterized by a low rooting response, an increase in the rooting percentage was registered when the laser treatment eliminated the tissue up to phloem proximities. Analysis of the nodal position showed that bud location was a preferential place for AR formation independently of wounding treatment. In case of *Rosa* ‘Tan09283’, laser treatments did not reduce its high rooting capacity, but an apparent reduction in rooting quality due to an investment in tissue healing was observed when wounding reached deeper layers such as parenchyma and sclerenchyma. Results also showed a strong AR formation directly from wounded regions in case of *Rosa* ‘Tan09283’ specifically when the wound was located below the axillary bud. In conclusion, wounding by assisted-elimination of layers by laser can induce positive effects on AR formation of single-node stem cuttings of the rose if energy applied is able to expose phloem proximities, a longitudinal orientation, and relative position to the axillary bud are considered.

## Introduction

Asexual propagation by cuttings is based on the ability of a detached plant part to regenerate a new root system as a plant survival strategy. This natural process has been exploited in order to vegetatively propagate species of economic and ecological value, thereby maintaining the genetic traits of the mother plants. Cutting propagation is of increasing interest for rose being the most important ornamental crop worldwide, because of its advantages regarding time and labour. Adventitious root (AR) formation capacity depends on a multitude of factors such as genetic, physiological and abiotic factors among others (Bellini et al., 2014). The interaction of all these factors has given rise to cases where certain genotypes of interest have a low capacity for AR formation as described for roses (Nguyen et al., 2020). In order to understand and improve clonal propagation by stem cuttings, the AR formation process has been studied at the molecular, biochemical and mechanical level. New research have brought the possibility to identify, for instance, the different phases of AR formation (De Klerk et al., 1995), the biochemical and molecular signalling of this process (Bellini et al., 2014; Druege et al., 2019) as well as the tissue layers where the *de novo* root organogenesis takes place (Costa, 2002; Ahkami et al., 2013).

Since AR formation by cuttings is directly related to mechanical damage as a result of plant detachment, wounding seems to be an important factor to trigger cell reprogramming and the initiation of the AR process. A detailed knowledge of the different responses produced by the wounding could open up the possibility of understanding and optimizing the propagation of plants by stem cuttings. In the horticultural practice, procedures to manipulate wounded regions have already been practiced mostly empirically as a factor to enhance AR formation especially in hardwood species. In this sense, mechanical wounding of regions at the base of a cutting has been applied manually for decades usually together with the application of exogenous auxins (Hartmann et al., 2002). The first experiments regarding the role of wounds in woody cuttings were reported 80 years ago in which, stem cuttings which have been repeatedly sliced, showed an increase in rooting compared with uninjured controls (LaRue, 1941). A similar rooting response was observed with split-base cuttings of dormant apple rootstock cuttings in presence of exogenous auxin years later (Mackenzie et al., 1986). According to MacKenzie et al. (1986), if much more cambium was exposed to the wounding across the stem base, cambial cells formed callus and the number of ARs along the wounded tissue increased.

Nowadays, implementation of new mechanical wounding methods has been studied in different organs mainly of *Arabidopsis thaliana* (Savatin et al., 2014), but only recently, the application of laser-assisted single cell ablation has allowed a better understanding of mechanical stress factors and subsequent plant strategies at the local, regional and systemic level (Hoermayer and Friml, 2019). The precision of laser technology has been able to target the elimination of a single cell or small groups of cells from root tips. Results have shown an immediate wounding response of the target cell and its surrounding neighbors which has resulted in an instantaneous change in turgor pressure followed by a local increase of auxin signalling in wound-adjacent cells (Hoermayer et al., 2020). The use of laser as a marking tool in stem cuttings has already been reported in the recent years, mentioning advantages in marking of codes (Marx et al., 2013), as well as a reduction of the risk of diseases compared to wounds carried out by knives. Besides its potential, a deep study of wounding treatments by laser on woody cuttings has not been reported so far.

Taking into account the economic importance of roses, as well as the information obtained for this ornamental crop so far in the genetic and hormonal fields (Nguyen et al., 2020; Otiende et al., 2021), this study aimed to elucidate the effect of wounding on the stimulation of AR formation during the propagation of rose leafy stem cutting using laser-assisted-ablation as wounding tool. Due to the precision and replicability of laser treatments for cell and tissue removal, the current work addressed the application of laser to determine the effects of wounds at specific tissue layers and two different distribution patterns along the stem base. Finally, besides the intrinsic rooting capacity of the cuttings, the influence of the exogenous presence of auxins was also evaluated due to its practical application in horticulture.

## Material and methods

### Plant material

Three different rose genotypes, the easy-to-root cut rose cultivar *Rosa* ‘Tan09283’ (Registration name ‘Beluga’) and two difficult-to-root genotypes out of the rootstock cultivar *R. canina* ‘Pfänder’ (internal numbers R02-3 and R02-6) were used in the experiments. *Rosa* ‘Tan09283’ stems were ordered directly from the company Rosen Tantau (Uetersen, Germany). There, the mother plants were cultivated in a substrate mainly consisting of cocopeat (60%), to which perlite (30%) and coconut fiber (10%) were added. 100 g m^–3^ of Radigen was added to fertilize the plants at the beginning of the cultivation keeping an electrical conductivity of 1.6 to 1.7 mS cm^-1^. Plants grew at 18°C during the day and 16°C at night without a special humidity control in the greenhouse. The two *R. canina* ‘Pfänder’ genotypes were acclimatized plants from *in vitro* propagation. Rooted plants were carefully washed, planted into commercial peat substrate used for cutting propagation (Steckmedium, Klasmann-Deilmann GmbH, Geeste, Germany) and kept under a foil tent in the greenhouse for 2 weeks without fertilization. Thereafter, the tent was gradually opened and after 4 weeks the acclimatized plants were potted into the same growing medium. Later, plants were cultivated in our greenhouse at temperatures of 20 to 22°C during the day, and 17 to 20°C at night, relative humidity between 70% to 90%, and an average light intensity during the day of 630 µmol m^–2^s^–1^ (PPFD). At the end of the fourth month of cultivation under these conditions, long stems were harvested as the material for the laser wounding process.

### Parametrization of laser features: Energy density, laser beam profile and beam size

The parametrization of the marking process was carried out using a continuous wave CO_2_ Laser (BRM Pro 600, power 80 W, Netherlands) with assistance of a rotary axis (CNC 80 mm Chuck Rotation - 3 Phase, Ly. Group, China). An air compressor (Hailea ACO-500, China) was connected to the laser system to avoid laser absorption caused by burning, protecting the laser lens inside the laser head, and cooling down the laser area temperature (data not shown). The determination of key parameters of the laser system was primordial for a proper quantification of energy and reproducibility of data later on. Among the parameters to be determined were the laser beam profile, beam size, laser power and energy density. The laser beam profile provides information about the form of the cavity caused by the laser from a cross-section perspective. In our case, the profile used was a flat top beam profile which was characterized by leaving a more or less constant ‘flat’ penetration at the bottom of the cavity after marking. To check the laser profile performance, laser pulses, each with a duration of 1.3 ms, were placed consecutively along a tube of polymethyl methacrylate (PMMA) with 7 mm outside and 5 mm inside diameter. For visualization purposes, the geometry of the laser beam profile was evaluated in this way due to the transparency of the PMMA in comparison with biological samples. To determine the beam diameter, the same arrangement of laser pulses was checked on the surface of *R. canina* ‘Pfänder’ and *Rosa* ‘Tan09283’ cuttings under a stereo microscope (Zeiss Stemi 2000-C, Germany). Regarding laser power, the laser equipment was commanded through the RDCAM 6.1 software, a program working with power percentages (application power/maximal laser power) as a reference unit. For this reason, the relation between laser percentage and maximal power in Watts was necessary for the calculation of energy density per unit area. Three different methods were tested to evaluate the power of the laser: a calorimeter (Model J22201, China), a Mahoney power meter (MAH-CYDF-100 CW, China), and Dohicky power meter (RDWorks Lab, England). Once the beam size, laser power and laser beam profile had been determined; the calculation of energy applied per unit area of one single laser pulse, also called laser energy density, was quantified with the following equation:


(Eq. 1) $${\text{ E}}_{\text{ d}}=\;\frac{{\text{P}}_{\text{ l }}}{{\text{A}}_{\text{ b }}}*\text{t}$$

Where:

E _d_: Energy density of laser (J cm^-2^)
A _b_: Area of laser beam (cm^2^)
P _1_: Power of laser (W)
t: Time of laser pulse (s)

Once the calculation of the energy of one single pulse has been determined, an arrangement of dots placed consecutively along the cutting surface was used for the design of the marking patterns. Based on previous histological analysis, the selection of three percentages of energy (6.0%, 6.5%, 7.5%) were used for the calculation of energy density.

### Set up of laser wounding experiments with rose cuttings

Since the idea of this work was to study the influence of laser wounding on AR formation in detail, three experiments were carried out described in summary in **Table 1**.

**Table 1:** Setup and laser marking patterns on rose leafy stem cuttings of the three experiments.

Experiment	Laser pattern distribution	Genotypes	IBA
**1** **Two strips** **(one at bud side and one opposite to bud)vs** **two rings** Three laser power percentages: (6.0%, 6.5%, 7.5%)	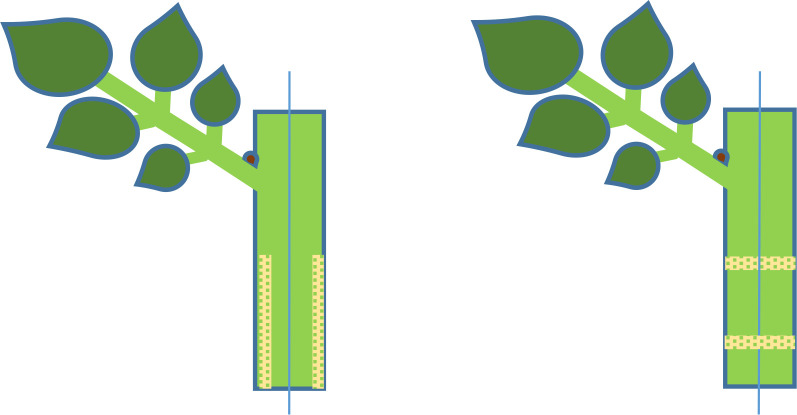	*R.* ‘Tan09283’ *R. canina* ‘Pfänder’ R02-3 and *R. canina* ‘Pfänder’ R02-6	Presence and absence of IBA (1 mg L^-1^)
	**Marking dimension (mm)**		
	*Rosa* 'Tan09283': Each strip: 15.0 x 1.7 Each ring: 15.0 x 1.8		
	*R. canina* Each strip: 14.0 x 1.7 'Pfänder' Each ring: 14.0 x 1.8		
**2** **One strip** **(at bud side)** **vs** **one ring** Two penetration levels: sclerenchyma and phloem	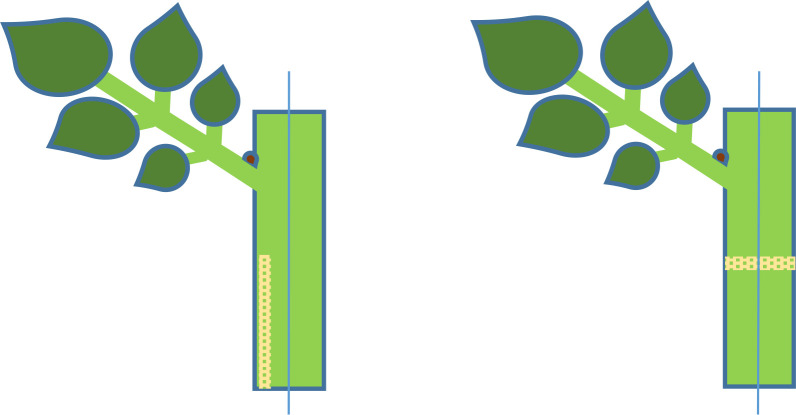	*Rosa* ‘Tan09283’ *R. canina* ‘Pfänder’ R02-6	Absence of IBA
	**Marking dimension (mm)**		
	*Rosa* 'Tan09283': 13.5 x 1.8 (for both patterns) *R. canina* 'Pfänder': 12.0 x 1.7 (for both patterns)		
**3** **One strip** **(at bud side)** **vs** **one strip** **(opposite to bud)** One penetration level: phloem proximities	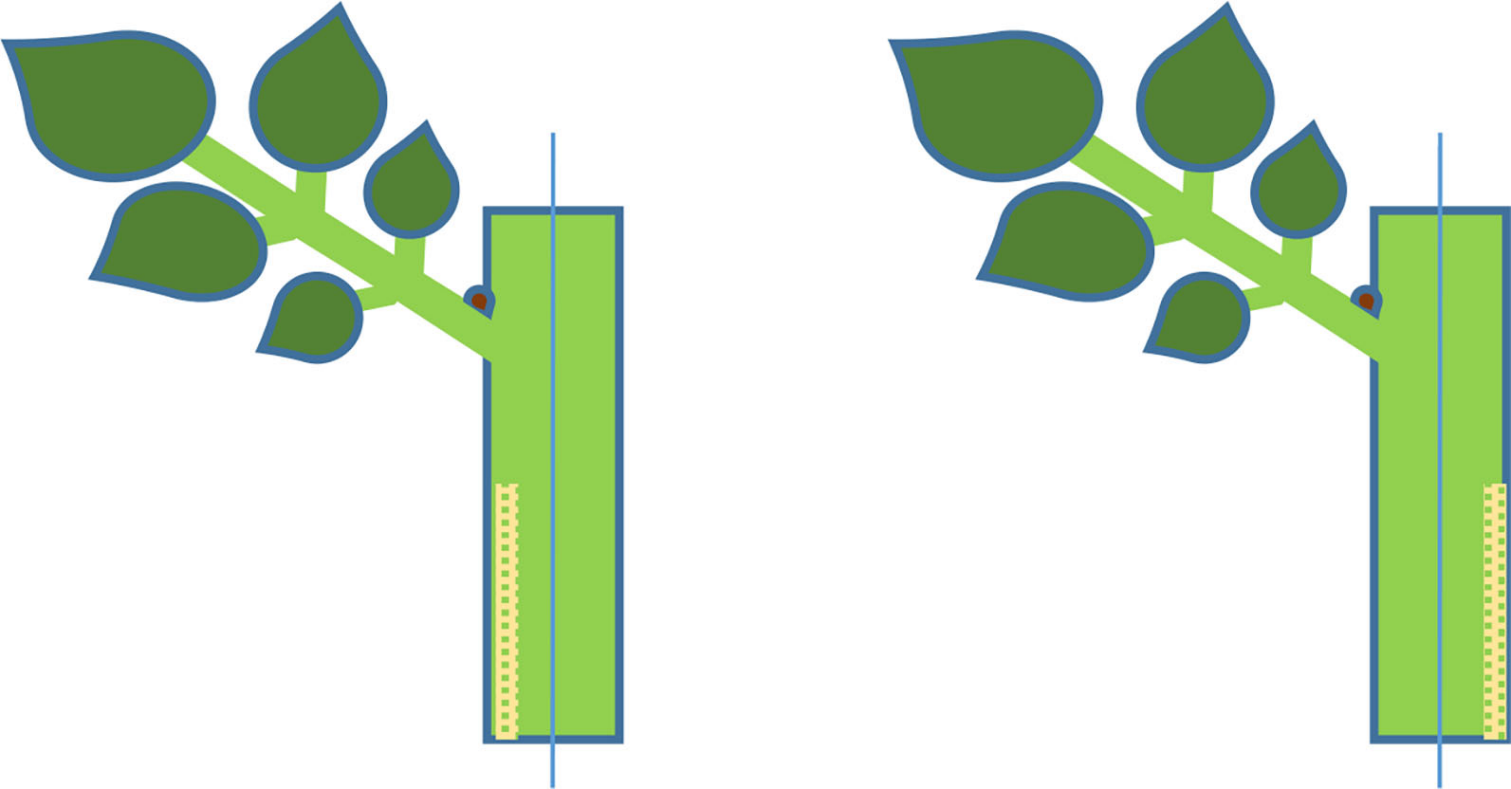	*Rosa* ‘Tan09283’	Presence of IBA (1 mg L^-1^)
	**Marking dimension (mm)**		
	Each strip: 16.5 x 1.8		

Yellow regions represent laser wounded areas distributed around or along the cutting base. The average cutting length was about 4 to 5 cm.

Experiment 1: The aim of this experiment was to determine the influence of energy applied on AR formation in general terms. As material, cuttings of *Rosa* ‘Tan09283’ and *R. canina* ‘Pfänder’ (R02-3 and R02-6) were tested applying three constant energy percentages (6.0%, 6.5%, 7.5%) distributed in two different marking patterns: two bands around the cutting (ring pattern) and two bands along the cutting (strip pattern) as depicted in **Table 1**. In case of strips, one band was located directly on the side of the axillary bud and the other one opposite to it. For rings, two bands were marked covering the whole cutting stem keeping a distance of 6 mm between both. In addition, a wounding treatment was done manually using a blade of a hand pruner (FELCO F-2, Switzerland) by applying a light pressure to the cutting on opposite sides of the cutting similar to the pattern of strips. The idea behind this manual treatment was to study the effects of mechanical wounding only, i.e. without thermal effects and lack of specific tissue penetration. Finally, a group of cuttings without any laser marking application was tested as a control group. Regarding average cutting diameter, for *Rosa* ‘Tan09283’ it was 5.2 mm, while cuttings measured 4.4 mm and 4.1 mm in diameter for R02-3 and R02-6, respectively. The influence of wounded-tissue was evaluated in presence and absence of exogenous auxin (IBA 1 mg L^-1^) during the propagation process. The experiment was arranged in a randomized complete block design. For *Rosa* ‘Tan09283’, each treatment was arranged in four blocks, each consisting of 10 cuttings (in total 40 replicates/treatment). In case of *R. canina* ‘Pfänder’ R02-3 and *R. canina* ‘Pfänder’ R02-6, the number of blocks was also 4, each consisting of 5 cuttings (in total 20 cuttings per treatment).

During experiment 1, one sample of each treatment was also monitored in real time by a special system designed for the monitoring of AR formation. This system consisted of a photobox divided into two compartments separated by a transparent acrylic plate. The first compartment contained a hydroponic system with the rose cuttings submerged in water, while in the second compartment a LED strip (Renkforce, LED-Strip RGB 24 W, Germany) and a web camera (Logitech BRIO Ultra-HD, United States) placed on top of a conveyor belt controlled by a stepper motor (NEMA 17-03, China) was installed. The whole system was connected to an Arduino Uno controlled through Halcon software version 13.0.4 and worked with the following duty cycles: first, the air compressor (Hailea SC438, China) of the hydroponic system worked continuously in dark conditions; second, the compressor was turned off and the LED strip turned on; third, the conveyor belt started moving until the camera was in front of a cutting. Once the camera had taken a picture, it moved to the next cutting driven by the stepper motor. Light remained on until the camera took a picture of every cutting (16 in total), whereafter the air compressor started again. This cycle was repeated every 2 hours and lasted around 2 minutes. A register of the cutting base images was compiled to a time-lapse video after 28 days of culture.

Experiment 2: The aim of this experiment was to determine the effect of laser wounding on the rooting response under a specific tissue layer exposition. In this case, cuttings of *Rosa* ‘Tan09283’ and *R. canina* ‘Pfänder’ R02-6 were marked using one ring and one strip pattern in absence of exogenous auxin. The strip pattern was positioned aligned to the axillary bud. The diameters of the cuttings were around 4.2 mm and 3.5 mm for *Rosa* ‘Tan09283’ and *R. canina* ‘Pfänder’ R02-6, respectively. Regarding tissue exposition, two different layers were addressed: sclerenchyma layer and phloem. In order to wound specific tissue layers for each cutting, a model based on the applied energy and cutting diameter was designed. Using the three constant energy percentages already mentioned in experiment 1 (6.0%, 6.5%, 7.5%), rose cuttings of different diameters (2.20 mm, 3.12 mm, 4.10 mm and 5.02 mm) were treated with both marking patterns. The evaluation of penetration and tissue layer reached was carried out by histological analysis based on 5 replicates per energy level. As for the result, the differences in tissue penetration for each cutting diameter were calculated, and trend lines were estimated to determine the specific level of energy needed to reach the different layers depending on cutting diameter. Additionally, the calculation of the maximum amount of energy that rose epidermis can withstand before tissue removal was determined. This parameter is known as laser induced damage threshold (LIDT), and was determined using cuttings of *Rosa* ‘Tan09283’ and *R. canina* ‘Pfänder’ R02-6 subjected to an additional series of energy percentages from 5.5 to 7.0%. The evaluation of injury was observed under the microscope and data was analyzed using the Weibull function. The experiment was arranged in a randomized complete block design. For *Rosa* ‘Tan09283’, each treatment was arranged in five blocks consisting of 10 cuttings (in total 50 replicates per treatment). In case of *R. canina* ‘Pfänder’ R02-6, each treatment was arranged in four blocks with 10 cuttings (in total 40 cuttings per treatment).

Experiment 3: In this experiment, the effect of laser wounding regarding its relative position to the axillary bud was evaluated in *Rosa* ‘Tan09283’ cuttings. Cuttings of this cultivar were marked using one single strip in presence of exogenous auxin (IBA 1 mg L^-1^) at two different positions: aligned directly below the axillary bud or opposite to the axillary bud. The average diameter of the cuttings was 5.3 mm. Regarding tissue exposition, proximities of phloem were reached based on the calculations for *Rosa* ‘Tan09283’ cuttings derived from experiment 2. The whole experiment was arranged in a randomized complete block design. Each treatment consisted of five blocks of 5 cuttings (in total 25 replicates per treatment).

### Rooting conditions

Rooting was carried out in two growth chambers under aeroponic conditions. Each aeroponic growing box (60 cm x 15 cm x 40 cm) was equipped with an independent water pump (Cadrim 25 W, China) with four sprinklers (Gardena 360°, Germany) and a capacity for 40 cuttings. Cutting bases remained in the air with support of a ring made of polystyrene while they were permanently irrigated with water with a pH of 5.5 to 6.5 and electrical conductivity of 0.18 to 0.24 mS cm^-1^. In case of experiments with exogenous auxin, a solution of 1 mg L^-1^ IBA was used instead of water. The following conditions were maintained in the growth chambers: temperature of 20 to 23 °C, relative humidity of 80 to 100% provided through an ultrasonic water nebulizer running 10 min every hour, and an average light intensity of 630 µmol m^-2^ s^-1^ (PPFD) 14 hours a day provided by sodium lamps (Philips 400 W, The Netherlands).

### Evaluation of AR formation and statistical analysis

Evaluation of AR formation was done from different perspectives depending on the aim of the study. One advantage of the aeroponic system was that it enabled a dynamic monitoring of each cutting in terms of number and position without direct manipulation. For every experiment, AR formation was evaluated until 28 days after planting (DAP) as described below. All statistical analyses were performed using the statistical software R 3.3.0 (R Core Team).

Experiment 1: Root presence was evaluated in quantitative and qualitative terms. In case of root presence, this aspect was divided in two different scenarios: basal and acrobasal rooting. Mean values and confidence intervals were calculated for rooting response. A correlation analysis between rooting rate and cutting diameter was carried out in this phase to check the possible effects of stem size during rooting. Additionally, a quantitative rooting evaluation was carried out. For this evaluation, the number of roots per rooted cutting and the fresh mass of roots were studied. The quantification of ARs was based on both parameters due to their simple and acquisition. In this sense, the number of roots per rooted cutting provided data regarding the amount of AR induced in the wounded zones, while the root fresh mass provided information about the average growth of the entire root system. For the quantification, cuttings were split longitudinally into halves with one half containing the leaf and the axillary bud. From each half, data of fresh and number and length of all roots were collected and expressed as mean values with standard errors (SE). Besides this manual evaluation, an algorithm done by Halcon version 13.0.4 was designed to compare the root mass of each side of the cutting based on pixel quantification and the real root mass. To quantify the relation between root mass and pixels, a photo of one side of each cutting was taken before the manual quantification.

Experiment 2: Rooting percentages were expressed as mean values and confidence intervals. Additionally, data of the position of every AR was collected and plotted in a polar coordinate system which represent the sum of all roots per treatment classified by the region from which they emerged. In addition to these diagrams, data of the average root number per rooted cutting was quantified.

Experiment 3: Rooting was evaluated in terms of root number and root fresh mass (mean values with SE). Additionally, the root distribution was evaluated through polar histograms as described for experiment 2. Finally, the dynamics of AR was determined based on a daily record of root formation throughout the whole propagation process.

Depending on the data distribution and homogeneity of residuals of the parameter analyzed, data were adjusted to analysis based on a linear model followed by analysis of variance (ANOVA). In case of lack of variance heterogeneity, data were transformed to a logarithm base and fit to a generalized linear model followed by a deviance analysis (F-Test). In case of significance, mean values were compared using Tukey’s multiple comparison test (p  ≤  0.05). Additionally, the correlation between root presence and cutting traits (cutting diameter and cultivar) was calculated employing Pearson`s rank correlation coefficient (r).

### Histology

Cross sections of rose stems of different diameters (2 mm, 3 mm, 4 mm, 5 mm and 6 mm), thus also representing different positions at the shoots, were histologically characterized in terms of bark anatomy. Additionally, cross and lateral sections of wounded cuttings were studied applying the laser energy percentages (6.0%, 6.5%, 7.5%) of experiment 1. Finally, an additional laser treatment at energy percentage of 8.5% was analyzed to represent the laser wounding at phloem proximities.

Histology study was performed based on a protocol published by Hoenemann et al. (2010). First, sections of cuttings were fixed in AFE solution (76% ethanol, 1.8% formaldehyde, 20% water and 5% glacial acetic acid) at 4°C for 24 hours. Dehydration involved a series of ethanol solutions (70%, 80%, 90%, and 96%) under vacuum. Then, samples were immersed in xylol followed by a paraffin/xylol (1:1) solution and finally infiltrated in pure melted paraffin (J.T. Baker, Deventer, The Netherlands). Samples were sectioned using a microtome (Leica 2065, Germany) with 8 μm thickness. Thereafter, all samples were stained with FCA solution according to Etzold (New Fuchsine-Chrysoidine-Astra blue: 1000 ml H_2_O, 0.1 g New Fuchsine, 0.143 g chrysoidin, 1.25 g Astra blue and 20 ml glacial acetic acid) (Morphisto, Frankfurt, Germany) for approximately 4 min. The sections were analyzed with a light microscope (Olympus BX61, Japan) using a camera (ColorView ll CCD Camera, Germany) and analyzed with Cell* imaging software (Olympus). For each sample, thickness of different tissue layers and laser penetration were measured, and the tissue layer reached by laser was identified.

## Results

### Energy determination and beam analysis of laser equipment

**Figure 1:**
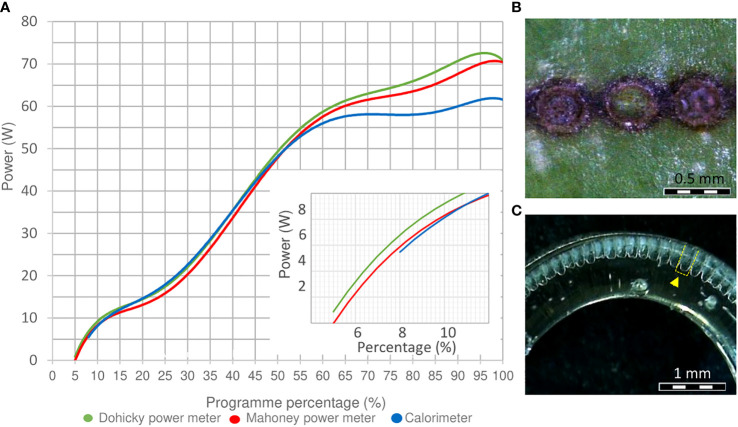
Power, beam size and beam profile determination. **(A)** Power determination of the laser equipment measured by a calorimeter J22201, a Mahoney, and Dohicky power meter. An enlarged section from 0 to 12% laser power is included at the corner of the diagram. **(B)** Beam size of a laser dot on the surface of a *R. canina* ‘Pfänder’ cutting. **(C)** Performance of a flap top beam profile (yellow marked) on a PMMA cylinder 7 mm outside and 5 mm inside.

The methods to determine the energy of the laser equipment showed a clear congruity in their results and pointed out that the laser power fluctuated in a non-linear way (**Figure 1A**). An increase in power started from energy percentages of 5.0%. Only the determination of power by the calorimeter presented differences in power starting from 60% compared to the two other power meters. Regarding the calculation of power used for experiment 1, percentages of 6.0%, 6.5% and 7.5% were equivalent to approximately 2.06 W, 3.13 W and 4.87 W, respectively. Regarding the diameter of the laser beam, the laser pulse generated a dot with an approximate diameter of 0.037 cm, and therefore, an area of 0.001074 cm^2^ (**Figure 1B**). Finally, the analysis of the laser beam profile showed that laser pulses generated relatively flat concavities in the center of each spot (**Figure 1C**). Based on this information and on Equation 1, the energy values per unit area of the power percentages of 6.0%, 6.5% and 7.5% were equivalent to 2.5 J cm^–2^, 3.8 J cm^–2^and 5.9 J cm^–2^, respectively.

### Rose stem anatomy and stem diameter

**Figure 2:**
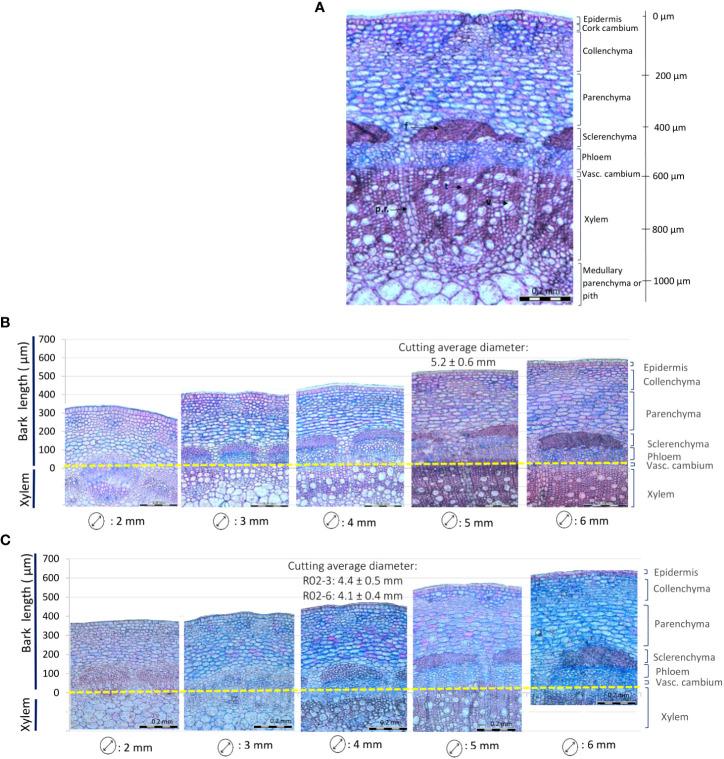
Rose stem anatomy depending on the diameter. **(A)** Partial cross section of a 5 mm rose stem of *Rosa* ‘Tan09283’. f: fibers, p.r.: parenchyma radial, t: tracheid, v: vessel. **(B, C)** Different stem diameters representing different positions on the shoot for *Rosa* ‘Tan09283’ **(B)** and *R. canina* ‘Pfänder’ **(C)**.

Histological analyses revealed that the bark of rose cuttings is composed by diverse groups of tissue layers interconnected in a thin region (**Figure 2A**). Starting from the outermost layer, the epidermis was a mono layer with an approximate size of 10 to 20 µm. Below this layer, the collenchyma and parenchyma were located, measuring approximately 100 and 150 µm, respectively. Both layers showed the presence of intercellular spaces and spherical and oval cell shapes. Then, the sclerenchyma, approximately 80 µm thick, was found to be a layer distributed in bundles slightly spaced around the stem. In this thin layer, the presence of lignin and narrow dead cells with thickened cell walls predominated. Just below, the phloem consisted of mostly conducting cells and had a thickness of about 100 µm. The vascular cambium was a monolayer of meristematic cells of smaller size and the xylem (approx. 300 µm) was composed of tracheids, xylem fibers and vessels. Finally, for stem diameters bigger than 5 mm, xylem was separated by a thin layer of radial parenchyma that elongated until connected with the pith.

Changes in the internal structure of the bark of *R. canina* ‘Pfänder’ and *Rosa* ‘Tan09283’ cuttings were observed with increasing diameters (**Figures 2B, C**). In both rose genotypes, 2 mm stems were characterized by soft tissue and absence of dead cells. In stems of 3 mm, secondary growth started resulting in xylem extension. Starting from 4 mm diameter, an increase in tracheids and vessels in the xylem layer took place, and the development of secondary cell walls in the sclerenchyma layer began. From 5 mm cutting diameter onwards, bark layers were fully mature, presence of fibers in the sclerenchyma was abundant and growth of radial parenchyma was evident. Cross sections with bigger diameters did not show major anatomical changes, except for the sclerenchyma layer, which became progressively denser.

### Histological analysis after laser wounding treatments

**Figure 3:**
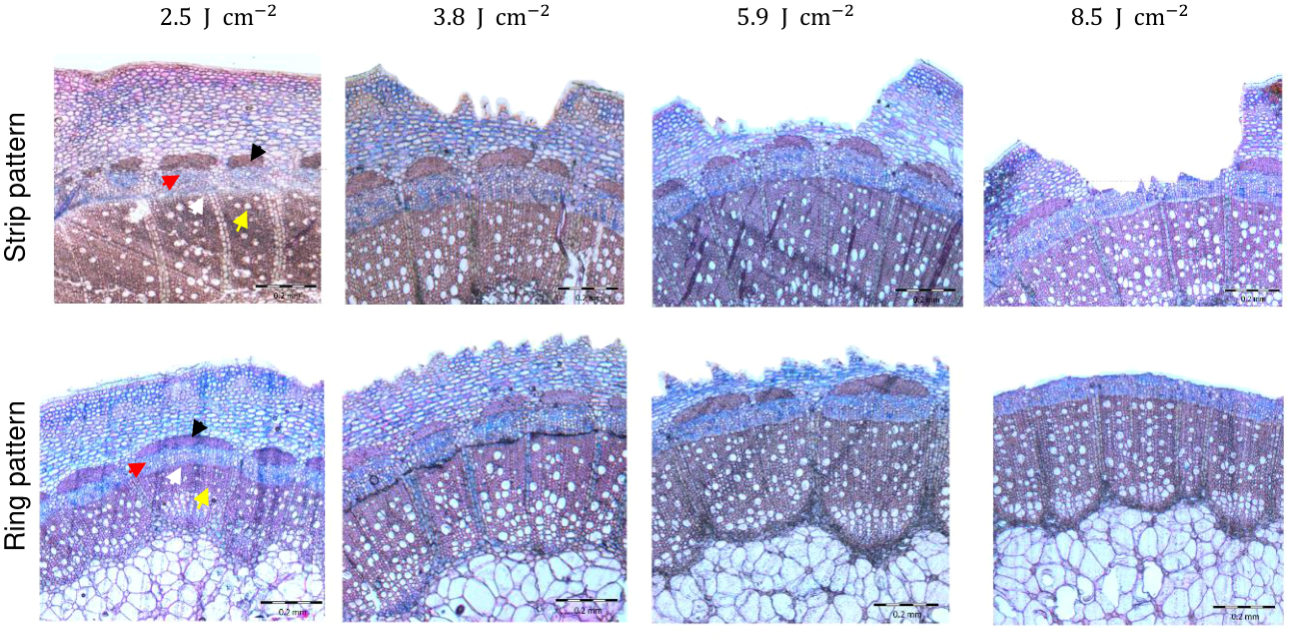
Histological analysis of laser wounding applied as strip and ring pattern on *Rosa* ‘Tan09283’ cuttings under four different energy densities (2.5 J cm^-2^, 3.8 J cm^-2^, 5.9 J cm^-2^ and 8.5 J cm^-2^). Arrows indicate sclerenchyma (black), phloem (red), vascular cambium (white) and xylem (yellow).

The analysis of cross-sections of the rose cuttings showed that laser penetration was progressive as result of the energy applied (**Figure 3**). The treatment with the lowest energy density of 2.5 J cm^-2^ did not result in visible tissue removal, but a slight change in color in the epidermis layer *in vivo*. Samples treated with this energy density showed light concavities on the epidermis probably due to dehydration induced by a thermal effect, but no cell collapse. Although laser marking treatments seemed to be slightly deeper for *R. canina* ‘Pfänder’ since they presented a thinner bark (approx. 544 µm) compared to *Rosa* ‘Tan09283’ (589 µm), there were no big differences in penetration between genotypes or laser patterns (**Table 2**). When an energy density of 3.8 J cm^-2^ was applied, several layers of tissue have been already removed including the epidermis, collenchyma and small parts of parenchyma. For *Rosa* ‘Tan09283’, the same energy density has led to a penetration of around 156 µm and 138 µm for the strip and ring pattern, respectively. In case of cuttings of *R. canina* ‘Pfänder’ R02-6, a bigger region of the parenchyma tissue was removed, corresponding to 190 µm in case of strip pattern, and 154 µm in case of ring pattern. Interestingly, the wounded surface after ring pattern application at 3.8 J cm^-2^ was not totally smooth during laser marking compared to strips. Finally, at 5.9 J cm^-2^ all layers above the sclerenchyma were removed, but some fibers of this layer were still present in some samples. In all cases, the presence of lignin was limited to the sclerenchyma layer and no other tissue layer besides xylem. To remove the sclerenchyma, an increase to an energy density of 8.5 J cm^-2^ was necessary and enough to leave the vascular tissue still present. An analysis of lateral histological sections showed that bands of sclerenchyma were still present after treatment (**Supplementary Material 1**). In addition to laser wounded cuttings, samples of cuttings wounded manually by a blade presented a high variability in wounding depth, making it difficult to reproduce (**Supplementary Material 2**). Overall, the average penetration (200 µm) using the manual method was similar to the laser treatment with 3.8 J cm^-2^.

**Table 2:** Penetration depth of laser wounding treatments (three different energy densities and manual wounding by blades) on rose cuttings depending the genotype (n = 5).

			Penetration depth (µm) under			
Genotype	Bark thickness(µm)	Pattern	Energy density_1_(2.5 J cm^–2^)	Energy density_2_(3.8 J cm^–2^)	Energy density_3_(5.9 J cm^–2^)	Blade method
*Rosa* ‘Tan09283’	585 ± 22	Strip	11 ± 16	156 ± 50	366 ± 32	210 ± 73
		Ring	10 ± 18	138 ± 52	340 ± 28	
*R. canina* ‘Pfänder’ R02-6	544 ± 29	Strip	16 ± 13	189 ± 27	388 ± 20	205 ± 76
		Ring	14 ± 13	154 ± 48	351 ± 46	

### Experiment 1: Rooting response after laser wounding using different laser energy densities in presence and absence of exogenous auxin

#### *R. canina* ‘Pfänder’ rooting response

**Figure 4:**
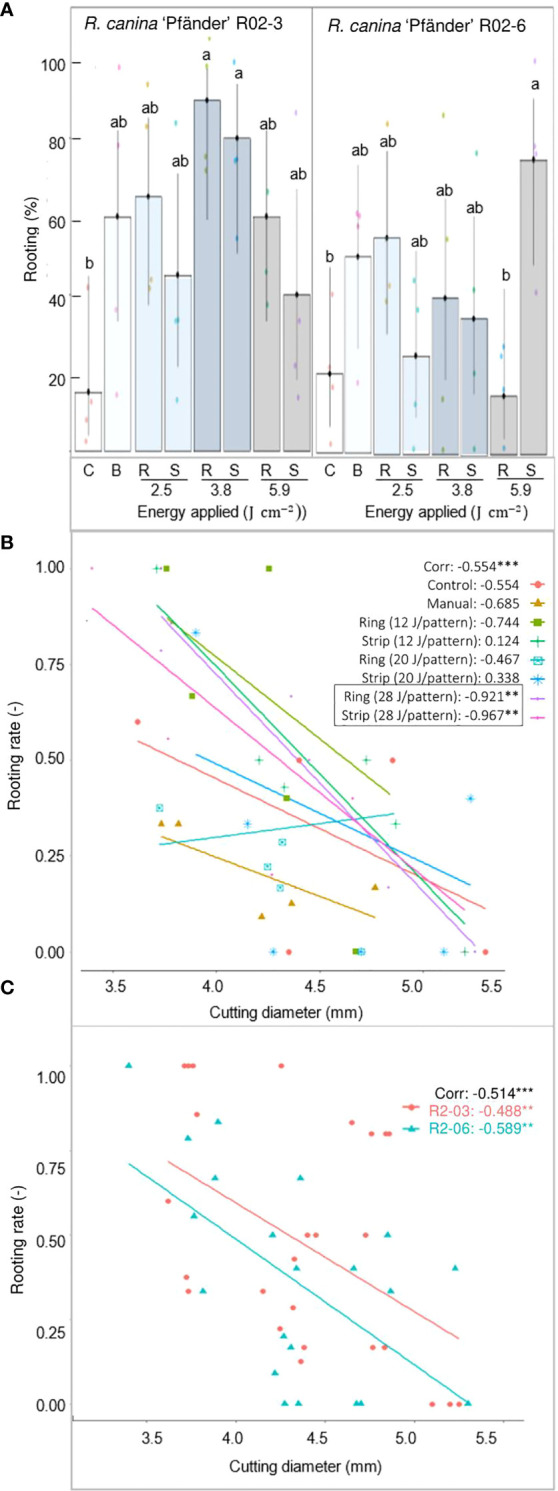
AR formation depending on the different wounding treatments of *R. canina* 'Pfänder' cuttings in presence of exogenous IBA (1 mg L-^1^) after 4 week of culture. **(A)** Rooting percentage of *R. canina* 'Pfänder' R02-3 and *R. canina* 'Pfänder' R02-6 classified by the treatment (three laser intensities and two laser patterns): C- control treatment, B- blade (manual wounding) treatment, R- ring pattern, S- strip pattern. Columns represent the mean values with its confidence intervals (n=20). Columns that do not share a common letter represent significantly different levels (Tukey test, p ≤ 0.05). **(B)** Pearson`s correlation between rooting percentage and cutting diameter classified by treatment. **(C)** Pearson`s correlation between rooting percentage and cutting diameter classified by cultivar. **, *** Significant at P ≤ 0.01, 0.001, respectively.

AR response was strongly enhanced by IBA for *R. canina* ‘Pfänder’ cuttings. Rooting percentages were below 20% in the absence of exogenous auxin, but reached up to 90% in its presence (**Figure 4A**). The overall very low rooting responses of the treatments without IBA did not allow the detection of significant differences among the treatments (**Supplementary Material 3**). However, was registered for wounded cuttings compared to the absence of rooting in the control. In the presence of IBA, only about 20% of the non-wounded control cuttings rooted compared to the higher rooting percentages in the treatments involving wounding. Due to very high rooting variation among cuttings, only two treatments showed significantly higher rooting percentages than the controls: the ring pattern with 3.8 J cm^-2^ which led to 90% rooted cuttings for R02-3, and the strip pattern with 5.9 J cm^-2^ which led to 76% rooted cuttings for R02-6. The correlation analysis between rooting rate and cutting diameter showed a clear influence of rose stem size on the AR induction. Interestingly, a negative correlation between AR formation and cutting diameter was detected in seven of the eight treatments evaluated (**Figure 4B**). Among them, only the ring and strip pattern with the highest energy density of 5.9 J cm^-2^ presented significance. A negative correlation was also observed between rooting rate and cutting diameter in terms of *R. canina* ‘Pfänder’ clones (R02-3 and R02-6) pointing out the higher rooting rate of cuttings with smaller diameters (**Figure 4C**).

#### *Rosa* ‘Tan09283’ rooting response

**Figure 5:**
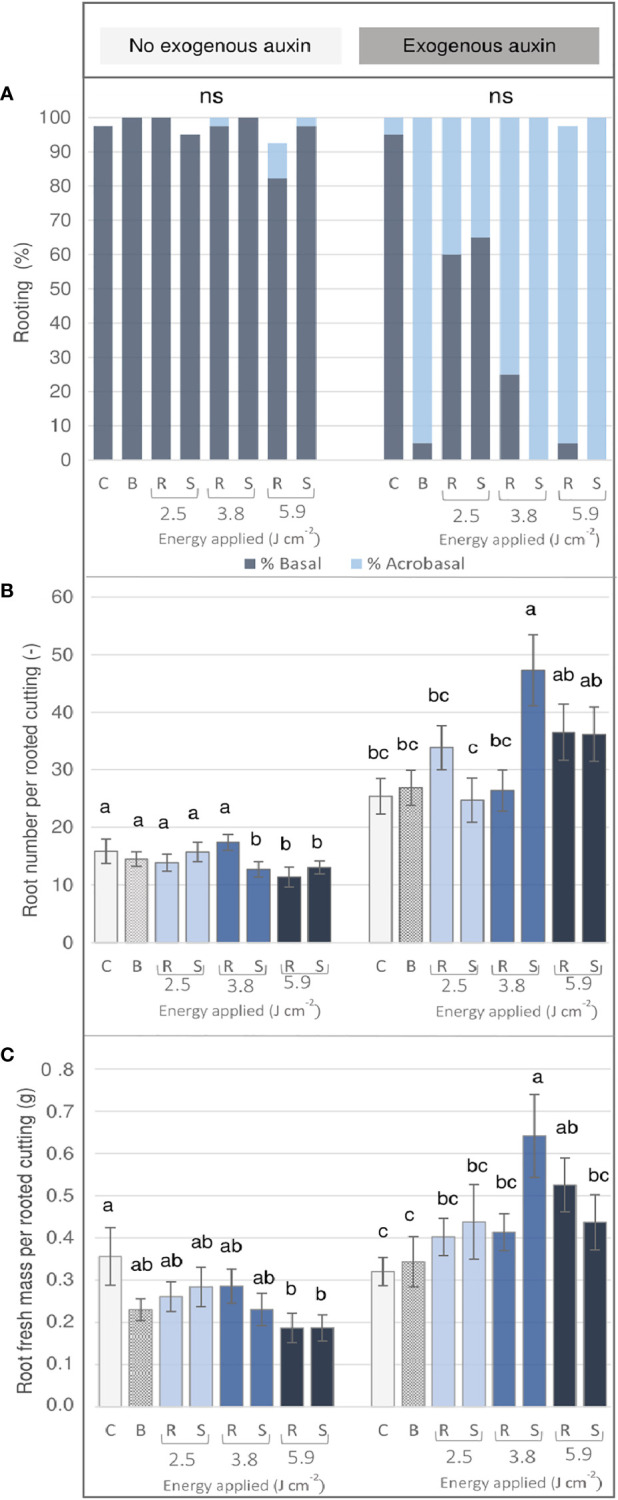
AR formation of *Rosa* ‘Tan09283’ cuttings depending on the treatment (three laser intensities and two laser patterns) in the presence or absence of exogenous IBA (1 mg L^-1^) after 4 weeks of culture. C- control, B- blade (manual) treatment, R- ring pattern, S- strip pattern. **(A)** Rooting percentage classified as basal and acrobasal rooting: Number **(B)** and total root fresh mass **(C)** per rooted cutting. Mean values and standard errors are shown at treatment level (n = 40). Columns indicated by the same letter, do not represent significantly different levels (Tukey test, p ≤ 0.05). ns, non-significant.

Cuttings of *Rosa* ‘Tan09283’ were characterized by a high capacity of AR formation which took place basally and acrobasally depending mainly on the energy density applied per pattern and the presence of exogenous auxin (**Figure 5A**). Basal rooting dominated in case that no exogenous auxin was applied, but also in the IBA treated control. Moreover, without exogenous auxin, a healing response of callus formation at the wounded areas was observed, especially at energy levels of 3.8 J cm^-2^ and 5.9 J cm^-2^ (data not shown). In contrast, in the presence of IBA, acrobasal rooting was observed not only directly at the wounded tissue at the two higher energy levels on both marking patterns, but also by the blade-made treatment. The quantitative rooting analysis showed two clear trends depending on whether or not auxin was added to the hydroponic solution. On the one hand, without IBA, both, the number (**Figure 5B**) and the fresh mass (**Figure 5C**) of adventitious roots were significantly reduced in laser treatments with the highest energy density of 5.9 J cm^-2^ compared to the control. On the other hand, in presence of exogenous IBA, the rooting parameters tended to increase as higher energy intensities were applied. Compared to the control, this increase was significant for the energy density of 3.8 J cm^-1^ by the strip pattern, followed by both laser patterns at 5.9 J cm^-1^. Interestingly in case of the manual treatment, no significant differences were reported compared to the control group.

#### Axillary bud position influenced distribution of ARs

**Figure 6:**
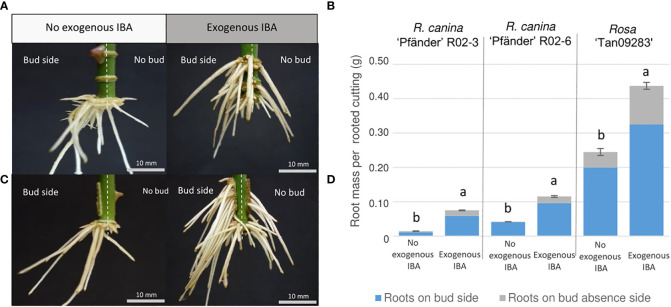
Adventitious root distribution regarding bud position by ring **(A, B)** and strip **(C, D)** patterns under the effect of exogenous auxin on *Rosa* ‘Tan09283’ cuttings. Left side: Stem cuttings are divided by vertical dashed white lines describing the division between the bud side and the opposite side of the cutting. Right side: Comparison of root mass ratio in presence and absence of exogenous auxin for *Rosa* ‘Tan09283’, *R. canina* ‘Pfänder’ R02-3 and *R. canina* ‘Pfänder’ R02-6. Columns and bars represent the mean values and SE (n= 20 for R. *canina* ‘Pfänder’ R02-3 and *R. canina* ‘Pfänder’ R02-6, n= 40 for *Rosa* ‘Tan09283’). Gray and blue sections in each bar represent the proportion of root mass on the cutting opposite to the bud or below the bud, respectively. Letters represent significantly different levels for each genotype (Tukey test, p ≤ 0.05).

Results showed that the axillary bud clearly influenced AR distribution independent of genotype. Interestingly, regions with the highest root number and root fresh mass were located directly below the bud also independently of treatment or presence of exogenous auxin. As an example, a comparison between both laser patterns at the energy density of 5.9 J cm^-2^ is illustrated for *Rosa* ‘Tan09283’ cuttings in **Figure 6**. In absence and presence of exogenous auxin, a ratio of approx. 75% of the root mass was located at the bud side despite big differences regarding quantitative rooting among both scenarios. Basal rooting was predominant for cuttings with a ring and strip pattern in absence of exogenous auxin, while an increment in acrobasal rooting was observed in presence of it. The Pearson`s correlation coefficients between the real root mass and the theorical root mass based on pixel analysis by Halcon showed a value of r blank = blank 0.74 suggesting that root mass determined by pixels was not completely quantified although root distribution ratios presented similar values (**Supplementary Material 4**). In case of *R. canina* ‘Pfänder’, acrobasal rooting was rarely found even in the presence of exogenous auxin. In contrast, root formation at the cutting base was completely suppressed and shifted towards the laser wounded areas in some *Rosa* ‘Tan09283’ cuttings under auxin presence.

### Visualization of AR formation after laser wounding

To register AR formation after laser treatments in more detail, photos were automatically taken to obtain time-lapse videos from selected cuttings of experiment 1. The development of the strip marking at 5.9 J cm^-2^ with and without exogenous auxin for *R. canina* ‘Pfänder’ R02-6 and *Rosa* ‘Tan09283’ is described as follows. For *R. canina* ‘Pfänder’, in the absence of exogenous auxin, callus formation was observed 4 days after planting (DAP) at the wounded regions, but AR formation was not achieved (**Supplementary Material 5**). In the presence of exogenous auxin, the *R. canina* ‘Pfänder’ cutting presented an accumulation of callus at the base of the cutting. After 21 DAP, the first root tip was visible at the base of the cutting just below the laser strips. Later on, more roots appeared and elongated rapidly without forming lateral roots (**Supplementary Material 6**). AR formation for *Rosa* ‘Tan09283’ showed the great rooting ability without the need for exogenous auxin. Callus started to develop from 4 DAP similar to *R. canina* ‘Pfänder’. Later, the first root tips emerged exclusively at the base of the cutting just below the laser marking where the largest amount of callus was localized 15 DAP (**Supplementary Material 7**). In the presence of auxin, from 3 to 10 DAP, callus growth was limited to the laser marked regions while no major change was observed at the base of the cutting. At 16 DAP, the first root tips were seen directly from wounded regions and also in adjacent zones resulting in a very high number of roots positioned on both sides of the cutting. At 21 DAP, the extension of some roots began while the growth of some others stopped. At the end of evaluation at 28 DAP, the root system on this particular case was composed of a total of 40 ARs with an average length of 1.70 cm (**Supplementary Material 8**).

### Experiment 2: Establishment of specific tissue layer exposition by determination of energy density depending on cutting diameter

**Figure 7:**
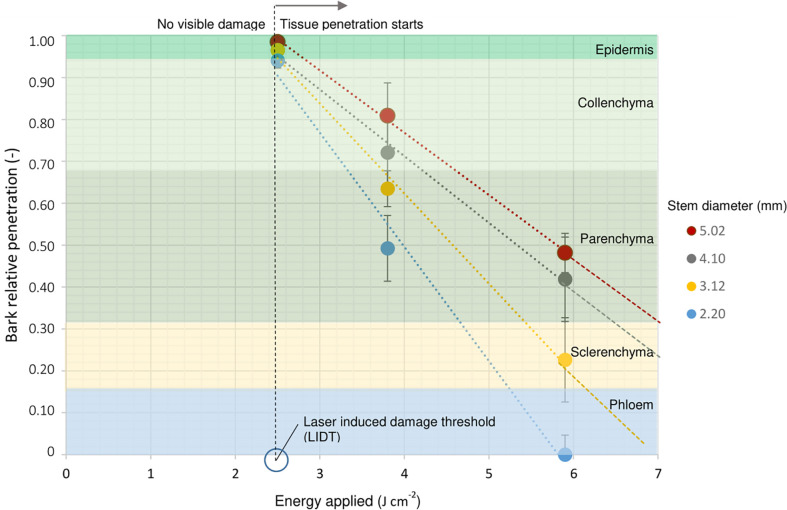
Laser penetration relative to the bark thickness in terms of energy applied for *R. canina* ‘Pfänder’ R02-6 cuttings depending on stem diameter. The different tissue layers that compose rose bark are represented in different colors and arranged in proportion to their original dimension in the bark. The vertical broken black line describes the laser induced damage threshold (LIDT) determined at 2.5 J cm^-2^. Dots represent the penetration mean values with SE, n = 5.

In order to expose specific tissue layers for each cutting, a model based on the laser energy applied and the cutting diameter was designed. Since cuttings with a smaller diameter require less energy than thicker cuttings to reach the same layer of tissue, the layer of tissue reached is directly proportional to the energy applied and inversely proportional to the diameter of the cutting. Results from this wounding analysis were expressed in relation to their relative penetration to compare all different cutting diameters (**Figure 7**). The only treatment where there was no difference in penetration independent of diameter, was that using of 2.5 J cm^-2^. The determination of the laser induced damage threshold (LIDT) for rose epidermis showed that the application of an energy density of 2.5 J cm^-2^ resulted in around 50% to 60% probability of epidermis damage (**Supplementary Material 9**). This means that this amount of energy was intense enough to ensure superficial tissue damage and reduce the risk of total tissue removal. Once the energy density increased to 3.8 J cm^-2^ and 5.9 J cm^-2^, differences in tissue exposition between cuttings became visible regarding cutting diameter. For example, the application of 5.9 J cm^-2^ on a 5-mm-cutting exposed only the collenchyma layer, while for 3-mm-cuttings the same intensity reached parenchyma regions. Likewise, in extreme cases for cuttings with 2 mm diameter, the application of an energy density of 5.9 J cm^-2^ could completely evaporate all bark layers, while it would only reach the parenchyma layer in stems of 5 mm in diameter. Due to overlapping of standard errors on penetration levels for 3.8 J cm^-2^ and 5.9 J cm^-2^ treatments, cuttings were classified based on the four trend lines obtained instead of developing a general mathematical model. Finally, trend lines were extrapolated to calculate higher energy levels in cases where the exposure of deeper tissue layers was needed.

### Rooting response after laser wounding under sclerenchyma and phloem exposition

**Figure 8:**
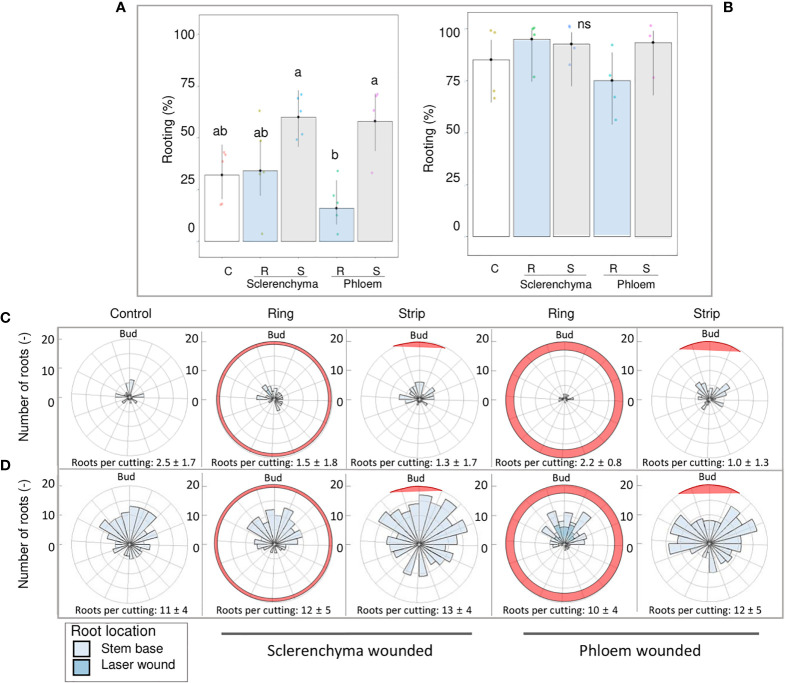
Comparison of ring and strip pattern laser treatments reaching defined tissue layers regarding rooting percentage and root distribution. AR formation of *R. canina* ‘Pfänder’ **(A)** and *Rosa* ‘Tan09283’ **(B)** cuttings when phloem and sclerenchyma layer were wounded without exogenous auxin. C- control treatment, R- ring pattern, S- strip pattern. Columns represent mean values and confidence intervals (n= 50 for R. *canina* ‘Pfänder’ R02-6 and n= 40 *Rosa* ‘Tan09283’). Root spatial distribution of the treatments for *R. canina* ‘Pfänder’ R02-6 **(C)** and *Rosa* ‘Tan09283’ **(D)**. Red shaded region represents the laser treatment position in relation to the axillary bud. Columns that do not share a common letter represent significantly different levels (Tukey test, p ≤ 0.05); ns, non-significant.

A comparison of laser patterns in *R. canina* ‘Pfänder’ cuttings showed a clear tendency of the strip pattern to increase rooting when sclerenchyma or phloem were reached (**Figure 8A**). In contrast, the ring pattern reaching phloem regions tended to reduce the AR percentage compared to the control. A reduction in the rooting percentages did not occur when the ring pattern only reached the sclerenchyma layer. In case of *Rosa* ‘Tan09283’ no significant differences in rooting percentage were detected nor high rooting percentages were reduced (**Figure 8B**).

A detailed analysis of the distribution of roots along the cutting based on polar histograms showed that both rose genotypes presented a similar root distribution in terms of treatment (**Figures 8C, D**). Results show a higher frequency of AR located on the axillary bud side. Moreover, large differences were observed in the total number of induced roots (counts) per region regarding genotype, and thus, in the number of roots per rooted cutting. As expected, the number of roots formed in *R. canina* ‘Pfänder’ R02-6 was lower compared to *Rosa* ‘Tan09283’. In case of control treatments, the number of roots per rooted cutting in *R. canina* ‘Pfänder’ was approximately 3 compared to 11 for *Rosa* ‘Tan09283’. For *R. canina* ‘Pfänder’, the number of AR per rooted cutting was between 1 and 2 in laser treatments. Due to an increase in rooting percentage with strips, the root counter for both histograms show an increase in spatial distribution of each treatment, even though AR per rooted cutting was similar. A common effect of the strip pattern reaching the phloem was a slight shift in root distribution to the sides of the strip for *Rosa* ‘Tan09283’ and *R. canina* ‘Pfänder’. Finally, for the ring pattern treatments particularly in case of exposed phloem, ARs were sometimes observed direct at the wounded area for both genotypes.

### Experiment 3: Effect of wounding and its relative position to the axillary bud on AR formation

**Figure 9:**
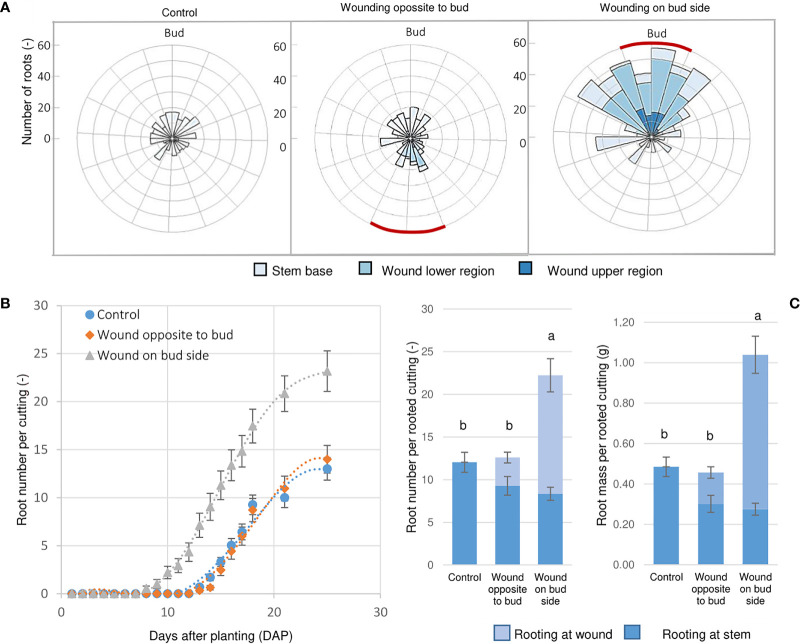
Wounding and bud position influence AR formation. **(A)** Root spatial distribution of *Rosa* ‘Tan09283’ cuttings when the sclerenchyma layer was reached applying a strip pattern on the side of the axillary bud and the opposite side in presence of 1 mg L^-1^ IBA. Red lines represent position of the laser strip treatment. **(B)** Dynamics of AR formation over time (days after planting) expressed as mean values with SE. **(C)** Root number and fresh mass distribution depending on the treatments. Columns represent the mean values and SE (n= 25). Columns that do not share a common letter represent significantly different levels (Tukey test, p ≤ 0.05); ns, non-significant.

Although the laser wounding promoted the development of ARs along the wound in *Rosa* ‘Tan09283’ cuttings in the presence of 1 mg L^-1^ IBA, large differences regarding root number were recorded depending on the wounding location (**Figure 9**). The rooting percentages were high for control and both laser treatments with 92% and 96%, respectively. However, each treatment presented particularities regarding the distribution of the ARs (**Figure 9A**). In case of control cuttings, the roots only originated basally in the previous experiments. When the strip pattern was located on the opposite side of the axillary bud, ARs formed sporadically in the lower region of the wound, but mainly at the base of the cutting. However, when the laser pattern shared the side of the axillary bud, a massive root growth was registered along the entire pattern and also at the cutting base.

Cuttings were analyzed daily to record changes over time regarding root number (**Figure 9B**). Interestingly, the strip treatment aligned to the bud not only resulted in higher root number and fresh mass, but it also induced AR formation earlier. In this case, roots were already observed 8 DAP. In contrast, if strip pattern was applied on the opposite side of the axillary bud, AR development became visible 14 DAP the same as the control treatment. By the end of the third week, the number of roots reached a plateau of about 22 AR per rooted cutting for the laser treatment below the bud, and 13 roots for the treatment opposite to the bud side and for the control. At the end of the experiment (28 DAP), significantly higher root fresh mass and number for the treatment with the strip aligned to the bud was recorded compared to the two other variants (**Figure 9C**). In case of the treatment opposite to the bud, although the total root number was not different from the control, their spatial distribution differed with some roots forming along the wounded areas.

## Discussion

### Bark size and cutting position affect not only tissue exposition to the laser treatment, but also AR formation in *R. canina* ‘Pfänder’

According to the histological results regarding rose stem anatomy, the different layers that compose the bark of rose cuttings showed a conserved morphology which grew proportionally to the diameter of the stem (**Figures 2B, C**). Nevertheless, differences in AR formation were given by variation in laser penetration levels due to the amount of energy density applied on cuttings with different bark thickness. Cutting diameter provided not only information regarding the maturity of tissues, the position where the cuttings were taken from, called the ‘topophysis’ effect, but also seemed to play a role during AR formation. This effect could explain why cuttings with smaller diameters presented higher rooting percentages compared to thicker diameters (**Figure 4C**). In accordance with this result, Otiende et al. (2021) also observed that cuttings in which nodal positions were closer to the shoot apex, rooted better and had a higher auxin–cytokinin ratio compared to basal cuttings.

The rooting response of *R. canina* ‘Pfänder’ in experiment 1, as a result of various levels of tissue penetration, showed that AR formation was strongly related to the exposure of a specific tissue layer. In this sense, the strong negative correlations between the cutting diameter and rooting rate at energy density of 5.9 J cm-2 for strip (r= −0.967) and ring (r = −0.921) patterns suggested that induction of ARs was higher at deeper penetration levels (**Figure 4B**), i.e. regions close to the vascular tissue (**Figure 3**). Each of the different tissue layers that composed bark has a certain structure and fulfills a specific role (Velada et al., 2021). Among them, the penetration of sclerenchyma, characterized by a high lignin content, was decisive for the wound response of AR formation. Overall, the fibers in the sclerenchyma provided effective protection against laser wounding treatments, suggesting that it is the main barrier protecting vascular tissue and cambium. According to Costa (2002), AR formation in single node leafy stem cuttings of rose occurs just below the sclerenchyma layer. In order to induce AR formation in rose cuttings, proliferation of undifferentiated cells between the cambium and the sclerenchyma results in root primordia that push through the sclerenchyma fibers to emerge (Costa et al., 2007). Future studies should include histological time series in laser-wounded cuttings in order to reveal spatio-temporal aspects of early phases of AR formation and to observe the fate of the founder cells. Interestingly, experiments in cuttings of *Corymbia torelliana* × *C. citriodora* also suggest that less lignification and sclerenchyma development may be related to improvements in AR formation (Wendling et al., 2015).

Another reason why AR formation in *R. canina* ‘Pfänder’ was highly dependent on tissue exposure could be related to differences in restorative rates after wounding of the different tissue layers (Marhava et al., 2019). Experiments applying laser wounding at the cellular level in tissue layers of roots, for instance, showed that cells in the cortex exhibited a lower rate of restorative cell divisions 12 h after laser wounding compared to the endodermis (Marhava et al., 2019). Similar results were reported when the meristematic activity of the pericycle cells was analyzed where only endodermis layer wounding triggered periclinal cell divisions whereas wounding of non endodermal root layers (xylem, cortex or epidermal cells) did not (Marhavý et al., 2015). In case of our single node laser-treated cuttings, not only restorative rates, but probably differences in the ratio of wounded area versus total stem surface area could also have an influence on the results obtained. Finally, and due to the differences regarding growth conditions of mother plants among *R. canina* ‘Pfänder’ and *Rosa* ‘Tan09283’, a comparison between both genotypes regarding AR formation or tissue reaction is not possible. In conclusion, since the initiation of cell division is an important stage of AR formation, a proper tissue exposition and area can also be expected to be the prerequisite to obtain reproducible wounding effects as well as to interpret AR formation in stem cuttings.

### Quantitative AR traits after laser wounding were drastically influenced by the presence or absence of exogenous auxin in *Rosa* ‘Tan09283’

Because *Rosa* ‘Tan09283’ cuttings did not present difficulties for AR formation, possible differences in tissue exposure did not affect rooting percentage to a greater extent as it was the case of *R. canina* ‘Pfänder’ R02-3 or *R. canina* ‘Pfänder’ R02-6. However, a progressive increase of tissue exposure depth gives rise to two completely different scenarios in quantitative terms depending on whether or not an external auxin supply was present. An increase in acrobasal rooting was observed as parenchyma, sclerenchyma and phloem regions were progressively exposed mainly when exogenous auxin was present (**Figure 5A**). Thus, an external auxin supply showed the ability to stimulate AR formation directly the wounded region when approaching the cell niches located in the layers of vascular tissue. Studies to determine the effect of auxins in the vicinity of the meristematic tissue in roots have shown that laser ablation has the capacity to launch the lateral root development program at the ablation zone only if endodermal cells, located next to the meristematic tissue, were reached (Marhavý et al., 2015). These results suggested that presence of exogenous auxins is capable of stimulating rooting (adventitious or lateral) if the ablation reaches the vicinity of meristematic tissue without the need to expose this tissue directly. Just in the case of *R. canina* ‘Pfänder’ cuttings in the presence of exogenous auxins, rooting position did not generally change from basal to acrobasal, nor an abundant increase in root number was observed. Therefore, a switch from basal to acrobasal rooting was not only related to tissue exposure or presence of external root stimulators, but also was strongly linked to the rose genotype.

A progressive increase in the tissue exposition led to a higher callus formation together with a reduction of fresh mass and number of roots in absence of exogenous auxins in *Rosa* ‘Tan09283’ cuttings (**Figures 5B, C**). Callus formation is a repair mechanism after wounding. Studies on laser ablation to determine tissue competence to initiate restorative cell division showed that after cell collapse, all cells were competent to initiate restorative processes even though the rate of cell restoration progressively decreased when moving away from the cell niches (Marhava et al., 2019). In case of cuttings, cell niches are located in the cambium and vascular tissue. It is precisely these tissues which are also competent for the formation of AR (Costa, 2002). Our results suggested that when deeper tissue layers were reached by the laser, such as parenchyma, sclerenchyma or phloem proximities, a higher induction of cell division was necessary to seal the wound, and therefore, a higher investment of resources for wound healing and less for AR formation was observed. Thus, due to necessary cell division for callus and AR, probably both processes competed for resources at deeper penetration levels. It is important to mention that callus formation is not necessarily a sign of AR formation (**Supplementary Material 10**), but rather a spontaneous response of cell repair (Marhavý et al., 2015). Interestingly, although an increase in callus formation was registered with laser wounding treatments, root emergence started at 14 DAP in all treatments, so no delays in root appearance due to wounding were registered. Studies by using auxin transport inhibitors such as kynurenine have shown the tissue healing process by callus development could still be carried out without the need of auxin long-distance transport or biosynthesis (Marhavý et al., 2015), and thus, other, as-yet unidentified auxin homeostasis processes, such as release of free auxin from conjugates, may play a role during tissue healing (Hoermayer et al., 2020).

### Axillary bud position causes AR formation mainly in the area aligned to it

The results obtained showed how the location of the bud directly influences the root positioning, making it a preferential place for AR formation (**Figure 6A**). It is crucial to analyse aspects related to the architecture of a leafy single-node stem cutting to understand the rooting results obtained. Since developing leaves and buds are strong sources of auxin (Garnett et al., 2010), differences regarding auxin source location between bud-side and non-bud-side of the stem were expected. The importance of auxin source and its influence on AR formation has been reported several times. Pacurar et al. (2014), for instance, were able to block the adventitious root formation in *Arabidopsis* removing the shoot apex (decapitation) from these plants. Studies suggested that auxin presence accelerates cell division and increased the sucrolytic activities at the base of the stem cuttings (Agulló-Antón et al., 2014). Therefore, the remaining leaf and axillary bud, including its outgrowth, became an important source of auxin which had to be transported basipetally to promote AR formation (Otiende et al., 2021). Moreover, not just the axillary bud, but also the remaining leaf contribute to the AR by providing biosynthetic carbon chains needed for cell healing and AR induction. Experiments regarding the influence of leaf area have proved, for instance, the influence of leaves on the growth of single node softwood stem cuttings of rose (Costa and Challa, 2002). Thus, this reinforces the explanation why AR was predominant in one side of the cutting as also depicted in **Figure 8**.

Interestingly, the effect of location of the axillary bud and leaf can potentiate AR induction if an exogenous source of auxin is present. Results showed that the alignment of the bud with the wounded tissue was essential for a massive increase in the number of adventitious roots (**Figure 9A**). In contrast, when the wounded tissue was located on the cutting side opposite to the bud, endogenous auxin and active resources maintained its flow towards the cutting base as main sink, while exogenous auxins were in contact with a section of phloem exposed in absence of an active auxin transport. The analysis of the rooting dynamics also revealed that source location and exogenous auxin at phloem exposition was able to accelerate AR induction six days earlier than in the other variants (**Figure 9B**). These results suggest that a stronger sink establishment was possible due to the interaction between endogenous and exogenous auxin directly at the phloem proximities, and thus, possibly accelerating the AR formation process.

### A specific tissue layer exposition by laser enables a proper study of AR regarding wounding

The analysis of laser wounding to different stem diameters indicated a clear relation between the applied energy and tissue exposure (**Figure 7**). Because the epidermis is the outermost tissue of the bark, this layer is subjected to responding to external forces and at the same time is under tremendous forces of turgor pressure from internal tissue (Landrein and Hamant, 2013). In mechanical terms, if the applied energy is less than the epidermal damage threshold, the damage caused does not produce irreversible effects on the tissue, maintaining its shape (Mirabet et al., 2011). In the case of single-node rose cuttings, the presence of permanent damage to the epidermis was determined from 2.5 J cm^-2^, which means that from this energy level, visible changes in the tissue *in vivo* took place. It is important to mention that although the histological analyses performed did not show clear mechanical damage to the epidermis layer, possible internal damage already existed due to protein denaturation processes or photochemical decomposition of organic compounds (Vogel and Venugopalan, 2003) especially in the cuticle of the cutting that has a water barrier function (Bourgault et al., 2020). A possible degradation of the cuticle components could explain why the presence of exogenous auxin during cutting propagation of *R. canina* ‘Pfänder’ R02-3 and *R. canina* ‘Pfänder’ R02-6 resulted in an increase in rooting percentages at 2.5 J cm^-2^ for both laser patterns (**Figure 4A**). Studies of laser ablation on partially injured tissue, i.e. without need for cell collapse, confirmed that exogenous auxin increased the expression of transcription factors such as ethylene responsive factor 115 (ERF115) in adjacent cells next to the wound (Hoermayer et al., 2020).

Even though our wounding pattern design was based on an array of dots with a duration of milliseconds (10^-3^ s), the energy exposure of the continuous wave CO_2_ laser was much higher compared to pulsed lasers. Analysis of laser interaction with living tissue showed that pulse laser exposure in the order of nanoseconds (10^-9^ s) triggers the first thermal effects on the surface of material marked (Mao et al., 2004). Compared to similar laser marking treatments done in rhododendron cuttings by Marx (Marx et al., 2013), the energy applied to reach vascular tissue in case of rose cuttings was approximately 3 times higher. This may be due to the fact that rhododendron cuttings were composed of more juvenile tissue, and due to the lack of sclerenchyma compared to rose cuttings. Nevertheless, due to differences on laser modus (continuous wave laser and pulsed laser) the comparability of these studies is limited. In this sense, to an adequate interpretation of data, the parametrization of laser features such as beam profile, power and dot size an adequate calculation of the energy density was quite relevant (**Figure 1**). Finally, due to seasonal variation specially for *R. canina* ‘Pfänder’, it is possible that also biological variation on the structure of rose cuttings could exist, and therefore, an analysis of wounding exposition and energy determination should be previously done with the material every time before marking. Additionally, and considering also biological variation among rose species, the calibration of tissue exposition should be specific for the species. Thus, a deeper study of the biological variation of rose cuttings morphology could led to the development of a more precise generalized model about tissue-specific laser exposition in future studies.

### The strip pattern positively influenced the AR formation compared to ring pattern when vascular tissue proximities were exposed

The first studies regarding the design of manual wounding patterns and its influence on sink establishment were carried out on pea epicotyls (Sachs, 1969) and later on bean seedlings (Gersani and Sachs, 1984). These experiments suggested that the establishment of resources basipetally such as auxin is done predominantly in the direction of the original polarity of the tissue in absence of injured tissue. This explains why active sources are accumulated mainly in the zone of the cutting base aligned to the leaves and bud. Nowadays, it is known that axillary bud must export auxin to be activated, and upon this activation, the process of auxin canalization begins (Domagalska and Leyser, 2011). Later on, AR process depends on the auxin transport establishment that can be non-polar and polar (Michniewicz et al., 2007). The non-polar auxin transport is fast, not saturable, and involves auxins to be loaded into the phloem and translocated along a concentration gradient, while polar auxin transport is relatively slow, saturable, requires energy investment, specific transport proteins and active free auxins (Michniewicz et al., 2007). Based on the differences on laser pattern used in our case, it is probable that a combination of both auxin transport systems occurred depending on the tissue layer exposed.

In case of a total interruption of basipetal transport at the phloem level as occurred in the ring pattern for *R. canina* ‘Pfänder’ cuttings, AR percentage tended to be reduced compared to the control group (**Figure 8A**). Interestingly in this treatment, AR formation took place directly at the wounded regions, suggesting that only the mechanical damage of the phloem around the whole cutting may drastically modify phloem-based transport. Additionally, it could be possible that a local stimulation of biosynthesis or release from conjugates may have been sufficient for minimum AR formation. The fact that some ARs were still formed at the cutting base after laser treatment as ring pattern pointed out the fact that auxin transport could still be carried out with only partial presence of vascular tissue where reestablishment of new vascular tissue could have been also necessary (Wulf et al., 2019). In contrast to rings, an increase in AR response by laser strip pattern when sclerenchyma and phloem regions were reached demonstrated that wounding pattern played a crucial role able to increase the AR induction of *R. canina* ‘Pfänder’ cuttings even though the mechanism behind it remained unknown (**Figures 8A, B**). In conclusion, laser pattern has an influence in AR percentage mainly if proximities of vascular tissue were exposed. In case of rings, this pattern has the potential to disturb the whole sink establishment of resources at this level of exposition, while for the strip pattern an increase in AR percentage was observed although rooting quality was sacrificed because of an investment of resources necessary for cell healing.

### Strip pattern positioned beneath the axillary bud enhanced rooting possibly due to auxin-jasmonic acid crosstalk in the wounded region in *Rosa* ‘Tan09283’ cuttings

Studies related to wounding in plant tissue suggest that stress conditions such as mechanical damage releases electric signals, ion fluxes, reactive oxygen species and wound-inducible hormones such as jasmonic acid (JA) (Savatin et al., 2014). So far, studies related to wound-inducible hormones confirmed an increase in the biosynthesis of jasmonic acid (JA) and ethylene (ET) in wounded stems of *Arabidopsis* (Asahina and Satoh, 2015). The idea that wounded tissue, and therefore an increase in jasmonic acid, can be related to AR formation was observed when applying JA in young cuttings of pea which also presented an early peak in IAA accumulation hours later (Rasmussen et al., 2015). Likewise, molecular analyses led to the conclusion that auxin is able to control AR formation initiation by a feed-backward crosstalk with the jasmonic acid homeostasis (Gutierrez et al., 2012). Based on these results, auxin transport models were developed in which auxin accumulation is triggered by its self-regulatory canalization linked to wound-induced biosynthesis of jasmonic acid (JA) and ethylene (ET) (Druege et al., 2016). We propose that the significant increase in AR formation by laser wounding in form of a strip pattern was possible because of an increase in JA signalling which interacted with auxin transported directly from the axillary bud, thus influencing the cross-talk between both phytohormones (jasmonic acid and auxin). Finally, it is important to mention that other hormones or sugars that are derived from the bud and adjacent fully developed leaf, respectively, could have potentially contributed to the predominant root formation on the bud side. Therefore, future biochemical analysis should be carried out to clarify the effect of wounding and its alignment to increase AR formation.

## Conclusions

This study has provided evidence that laser wounding based on the calculation of energy applied has the capacity to stimulate AR formation in leafy single-node stem cuttings of rose in tune with parameters such as axillary bud location, vascular tissue exposure and direction of marking. Regarding levels of energy, a parametrization of laser features was crucial to expose the specific tissue layers that compose rose bark. According to our results, values below 5 W were sufficient to generate wounds with different levels of exposure on the surface of rose cuttings. Moreover, the findings that different levels of tissue exposition generate a diverse reaction of AR formation highlights the importance of a proper exposure of phloem proximities by removal of sclerenchyma layer for AR induction. An improvement in rooting percentage in presence of a strip pattern shows the importance of a longitudinal wound sense along the cutting base. However, besides application of wounding treatments, stimulation of AR was highly influenced by genetic traits. Wounded tissue was widely influenced by the application of exogenous auxin, especially if the exposed tissue was directly aligned to the axillary bud. In this sense, the side where the axillary bud and leaf are located become a preferential place for AR formation due to synthesis of important active sources such as auxin, carbohydrates and possible other active components on these areas. Finally, the use of laser to generate wounds on the bark of leafy single-node stem cuttings opens up a potential tool to study AR formation depending on tissue or even specific cell exposition and distribution. Nevertheless, further studies at the biochemical, molecular and histological level are necessary to explain the effects of wounding on AR formation.

## Data availability statement

The raw data supporting the conclusions of this article will be made available by the authors, without undue reservation.

## Author contributions

RJMO took part on the laser parametrization, evaluation of experiments, statistical analysis and wrote most of the manuscript. TW contributed to the experimental setup, plant material supply, and supervised biological aspects of the research. AB contributed to the plant selection aspects and assistance regarding rooting evaluation. TR was involved in the analysis of image analysis as well on the engineering of the project and technical aspects. All the authors edited and approved the manuscript.

## Funding

This work was finantially supported by the research funding of the Hochschule Osnabrück, the Biosystem Engineering Laboratory (BLab) and the Section of Woody Plant and Propagation Physiology at the Leibniz Hannover University.

## Acknowledgments

We thank the reviewers for their insighful comments on this manuscript and all members of the Blab team at the Hochschule Osnabrück for help devising the imaging analysis process and the technical support during the design of the whole project. To all the members of the floriculture section of the Hochschule Osnabrück for the greenhouse facilities. We would like to thank all members of the Woody Plant and Propagation Physiology Section at the Leibniz University Hannover for the advice regarding microscopy and the supply of the plant material of *R. canina* ‘Pfänder’. Finally, a special thanks to Zahra Mohammadi Nakhjiri for the linguistic review of this article.

## Conflict of interest

The authors declare that the research was conducted in the absence of any commercial or financial relationships that could be construed as a potential conflict of interest.

## Publisher’s note

All claims expressed in this article are solely those of the authors and do not necessarily represent those of their affiliated organizations, or those of the publisher, the editors and the reviewers. Any product that may be evaluated in this article, or claim that may be made by its manufacturer, is not guaranteed or endorsed by the publisher.
