# Supplementary figures and images for: Stimulation of adventitious root formation by laser wounding in rose cuttings: A matter of energy and pattern
Source: Front Plant Sci. 2022 Sep 29;13:1009085. doi: 10.3389/fpls.2022.1009085 (PMC9557736; doi:10.3389/fpls.2022.1009085)

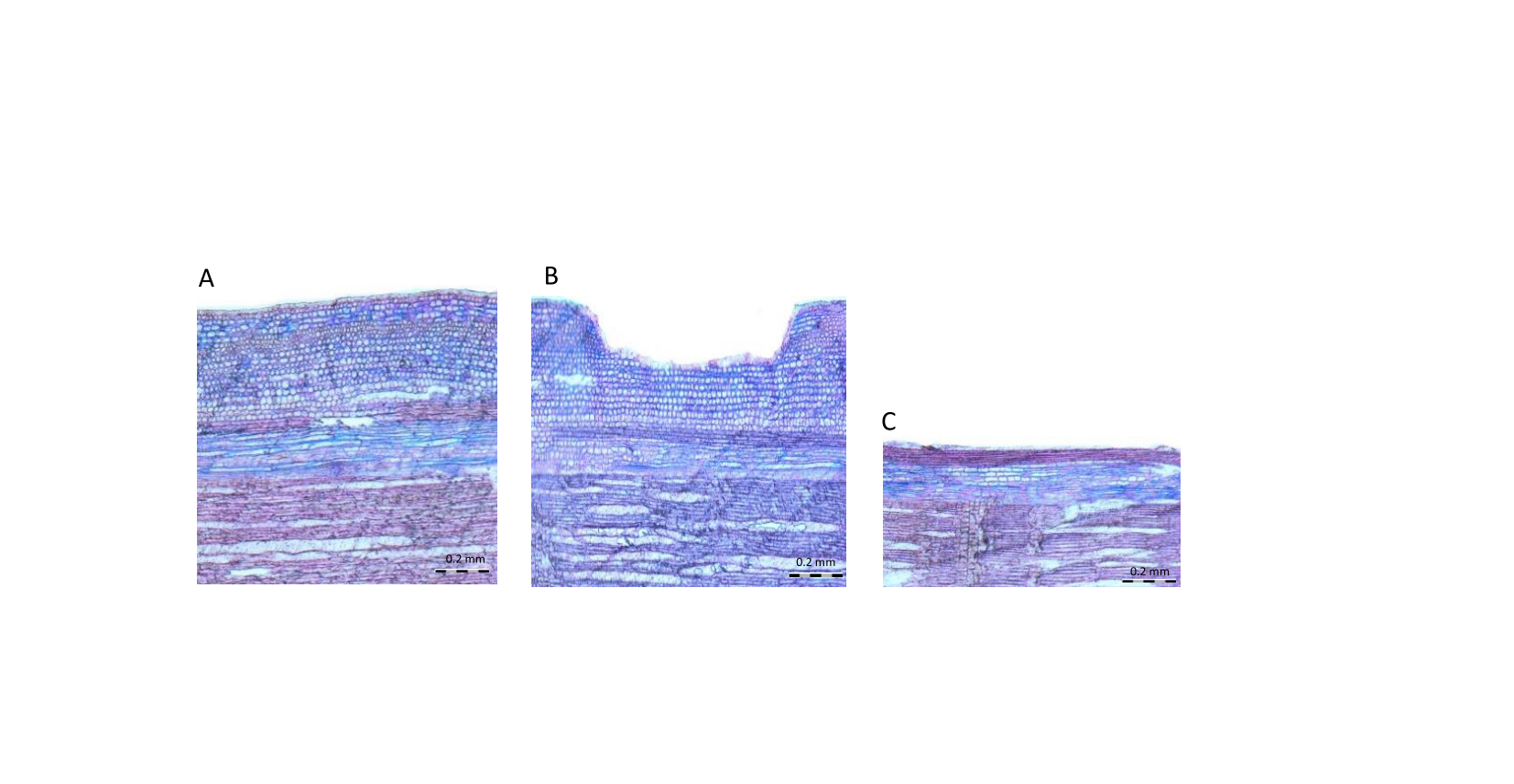

Supplement: Supplementary file 1 [file Image_1.tiff]

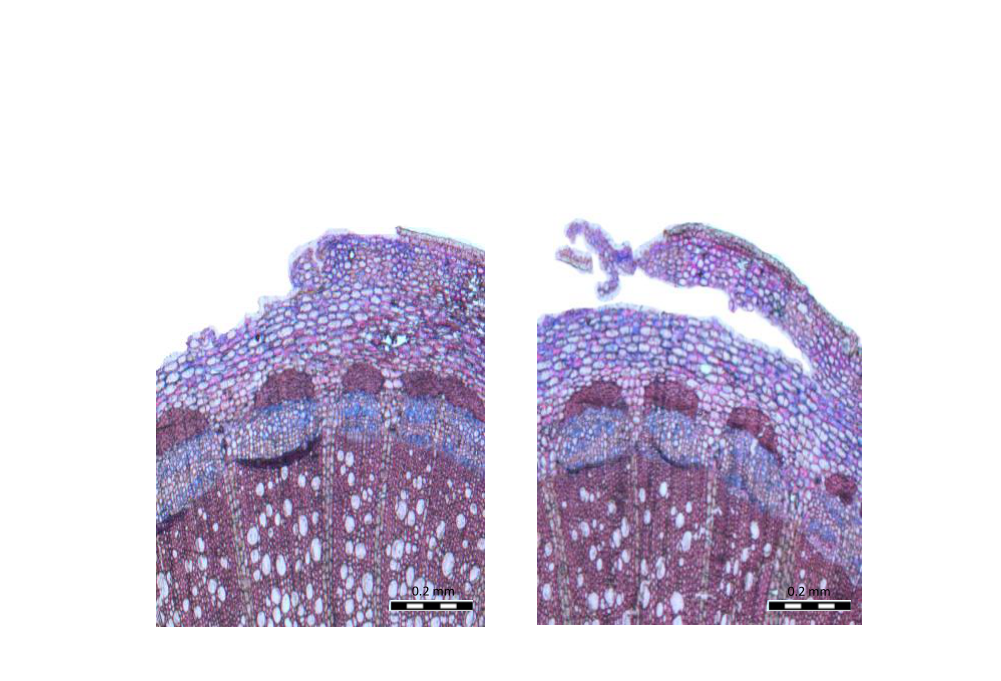

Supplement: Supplementary file 2 [file Image_2.tiff]

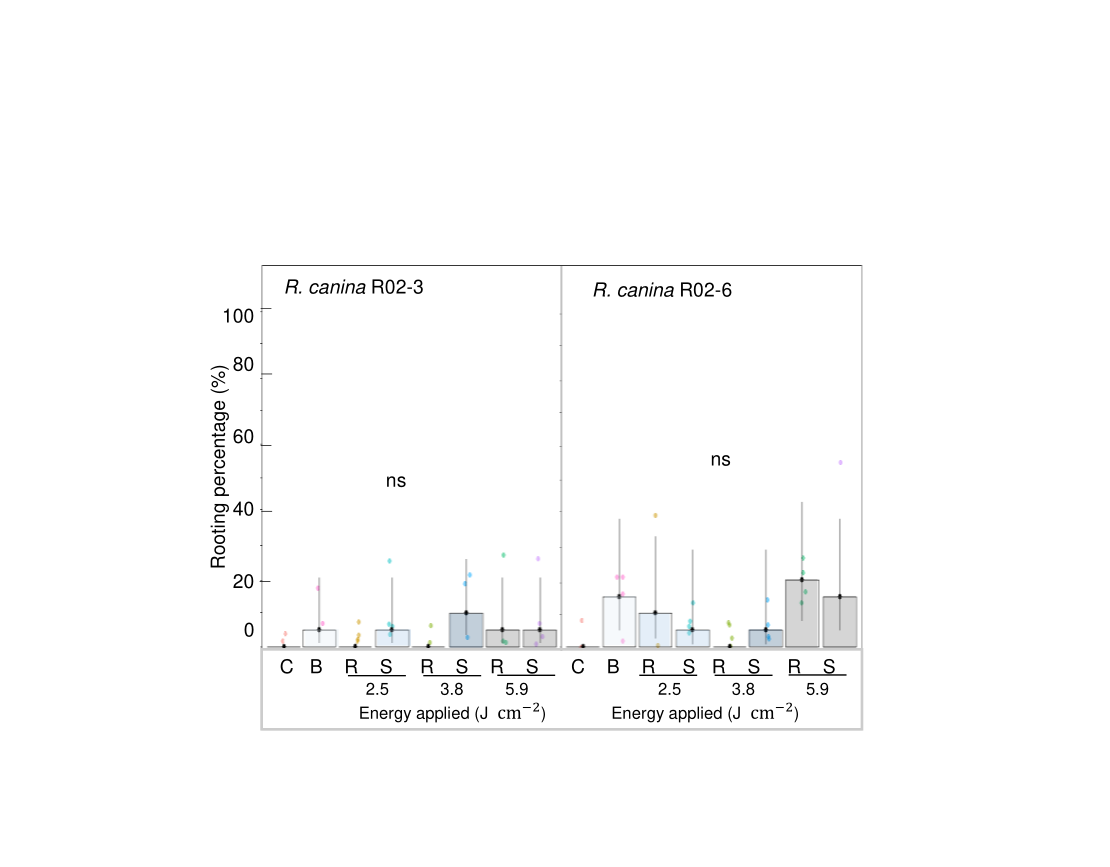

Supplement: Supplementary file 3 [file Image_3.tiff]

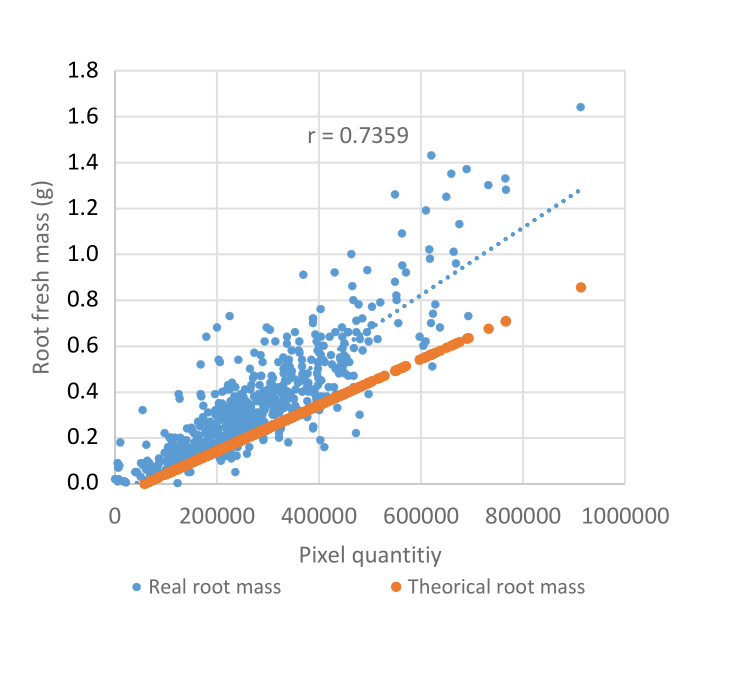

Supplement: Supplementary file 4 [file Image_4.tiff]

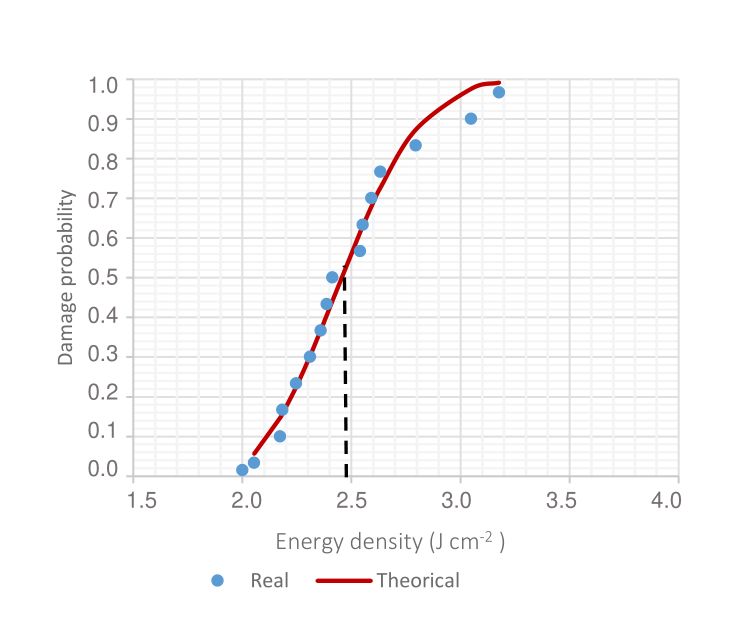

Supplement: Supplementary file 9 [file Image_9.tiff]

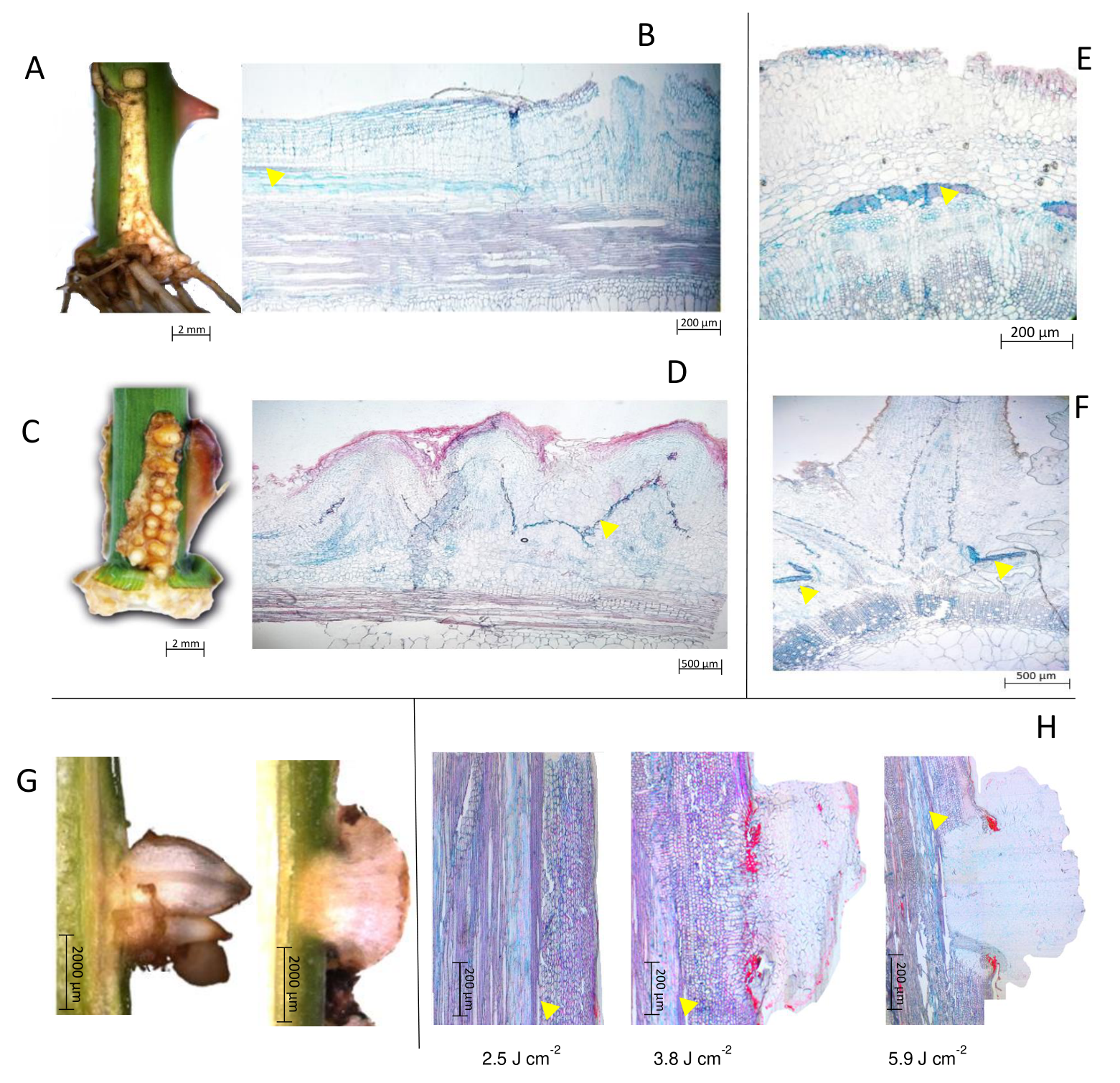

Supplement: Supplementary file 10 [file Image_10.tiff]
